# From conjugation to T4S systems in Gram‐negative bacteria: a mechanistic biology perspective

**DOI:** 10.15252/embr.201847012

**Published:** 2019-01-02

**Authors:** Gabriel Waksman

**Affiliations:** ^1^ Institute of Structural and Molecular Biology UCL and Birkbeck London UK

**Keywords:** bacterial conjugation, DNA and Protein Secretion, pilus biogenesis, relaxosome, type IV secretion system, Microbiology, Virology & Host Pathogen Interaction, Structural Biology

## Abstract

Conjugation is the process by which bacteria exchange genetic materials in a unidirectional manner from a donor cell to a recipient cell. The discovery of conjugation signalled the dawn of genetics and molecular biology. In Gram‐negative bacteria, the process of conjugation is mediated by a large membrane‐embedded machinery termed “conjugative type IV secretion (T4S) system”, a large injection nanomachine, which together with a DNA‐processing machinery termed “the relaxosome” and a large extracellular tube termed “pilus” orchestrates directional DNA transfer. Here, the focus is on past and latest research in the field of conjugation and T4S systems in Gram‐negative bacteria, with an emphasis on the various questions and debates that permeate the field from a mechanistic perspective.

**Glossary**
CP: coupling protein
cryo‐EM: cryo‐electron microscopy
Cryo‐ET: cryo‐electron tomography
dtr: DNA transfer and replication
ICE: integrated conjugative elements
IMC: inner‐membrane complex
IM: inner membrane
IR: inverted repeat
LPS: lipopolysaccharides
mpf: mating pair formation
OMCC: outer‐membrane core complex
OM: outer membrane
OmpA: OM protein A
RHH: ribbon‐helix‐helix
T4S: type IV secretion
TrIP: transfer DNA immuno‐precipitation

## Introduction

Bacterial conjugation is the process by which DNA is transferred unidirectionally from a donor cell to a recipient cell. It plays a crucial role in horizontal gene transfer, the major means by which bacteria evolve and adapt to their environment, and also a process of immense biomedical importance since conjugation is the main vector of propagation of antibiotics resistance genes. It was first described by Lederberg and Tatum in the 1940s 1. Its discovery signalled the dawn of molecular biology once it was demonstrated that the transfer of genetic information was unidirectional and that the entire genome of *Escherichia coli* could be passed from one cell to another starting at a defined site 2. Indeed, landmark discoveries followed: the mapping of the *E. coli* genome (mapped in “minutes”, i.e. the time taken by a particular gene to be transferred from donor to recipient, with time 0 being the mating start—when donor and recipient cells were put in the presence of each other) or the discovery of gene structure and regulation (please refer to the fascinating account of this research in the Nobel lectures by the founding fathers of the field of molecular biology, Francois Jacob, Andre Lwoff and Jacques Monod in 1965 3).

**Figure 1:**
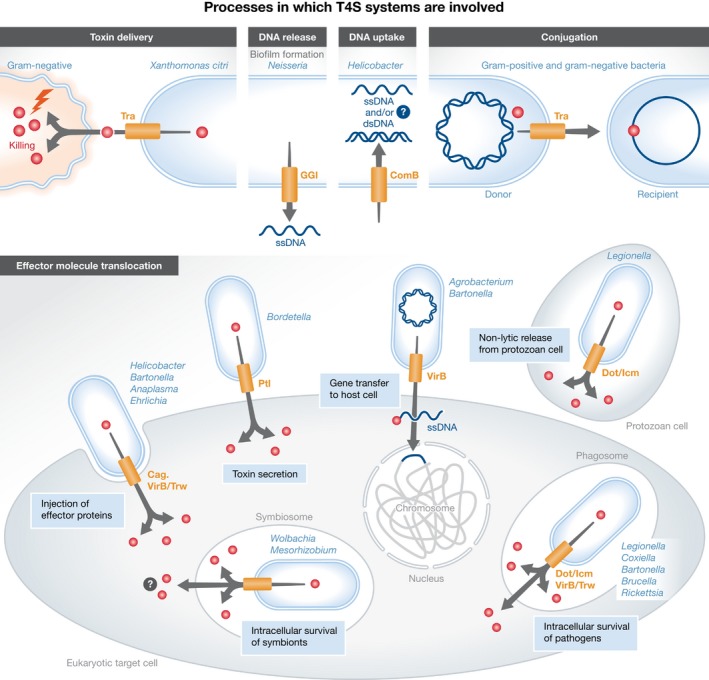
The various processes in which T4S systems are involved T4S systems are involved in DNA transport during conjugation, transformation and *A. tumefaciens* infection, and in effector transport by a number of bacterial pathogens. This figure was modified from Grohmann *et al* 108.

The various machineries utilized during conjugation to execute DNA transfer are usually encoded by conjugative plasmids or other genetic mobile elements such as integrated conjugative elements (ICE). Plasmids are ubiquitous in bacteria and are defined as a collection of genetic modules organized into a stable, usually circular, self‐replicating replicon, which does not usually contain genes essential for cell functions (reviewed in ref. 4). Several of these modules contain genes encoding proteins that assemble into large complexes mediating most commonly the plasmid's own transfer to a recipient bacterial cell, but also intriguingly (but rarely) to a eukaryotic cell such as yeast, plant or human cells 5, 6, 7. Interestingly, these modules are evolutionary related to clusters found in genomic islands of a restricted number of bacterial pathogens such as *Helicobacter pylori*,* Bordetella pertussis* or *Legionella pneumophila* where they play essential roles in pathogenicity by injecting protein effectors into eukaryotic hosts 8 (Fig 1).

Conjugation in Gram‐negative bacteria is mediated by three large complexes: a DNA‐processing machinery called “the relaxosome”; a membrane‐embedded transport machinery termed “type IV secretion (T4S) system”; and a pilus 9.

Conjugation starts with the assembly of the relaxosome to a particular site on the plasmid DNA called the “origin of transfer” or OriT. The relaxosome includes one key protein called the “relaxase” and a number of accessory proteins. The relaxase plays essential roles: (i) it catalyses a nicking reaction on a single strand of OriT DNA at the so‐called *nic* site and covalently reacts to the 5′‐phosphate generated by the nicking reaction; and (ii) it binds to the T4S system through interactions with one of the constituents of the transport machinery, the coupling protein (reviewed in ref. 10).

The T4S system is one of six secretion systems embedded in both membranes of Gram‐negative bacteria 11. Minimally, they are composed of 12 proteins termed “VirB1‐11 and VirD4” (to use the naming nomenclature derived from the *Agrobacterium tumefaciens* T4S system) 12. Three components, VirB7, VirB9 and VirB10, form the so‐called outer‐membrane core complex (OMCC), absent in Gram‐positive T4S systems where there is no OM 13. The OMCC connect to an inner‐membrane complex (IMC) composed of VirD4, VirB4, VirB3, VirB6, VirB8 and part of VirB10. OMCC and IMC are connected through a stalk of unknown composition, perhaps made of VirB2 and VirB5 14 or VirB10 14, 15, 16. At least two ATPases (VirB4 and VirD4), or sometimes three (VirB4, VirD4 and VirB11), power the system.

Finally, the conjugative pilus of Gram‐negative bacteria is an essential element in conjugation. For decades, it was the only feature in conjugating cells that could be observed or purified 17. It is made of a major component, VirB2, and a minor one, VirB5. VirB2 assembles into a large helical filament with perhaps VirB5 at its tip 18. Pili have been hypothesized to either serve as attachment devices mediating recognition of and attachment to recipient cells or serve as a conduit for relaxase/ssDNA transport, or both. Some conjugative pili are capable of retraction, which will bring donor and recipient cells together 19 resulting in close proximity. Indeed, tight conjugative junctions have been observed which have led to the suggestion that cell‐to‐cell contacts are required for conjugation to take place 20, 21. However, transfer has also been observed when cells are some distance apart (see detailed discussion below) 22.

In this review, I will first describe the up‐to‐date knowledge on each of these complexes and then will discuss the various and sometimes contradictory mechanistic insights that the most recent research shed on the mechanisms of conjugation and type IV secretion.

## The relaxosome

Excellent reviews have been written on the subject 10, 23, 24 and I will here only focus on recent research illuminating relaxase mechanism.

### Relaxases

**Figure 2:**
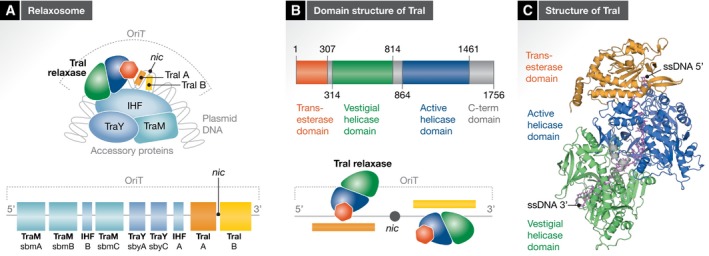
The relaxosome (A) Schematic diagram of relaxosome composition and assembly. Upper panel: Composition of the F plasmid family relaxosome. The F‐family relaxosome is composed of the relaxase TraI and three accessory proteins: TraY and TraM encoded by the plasmid and IHF encoded by the bacterial genome. All proteins assemble at the plasmid's origin of transfer (OriT) in a process that affects DNA topology around the OriT region. OriT also contains the nick site (*nic*). This site is flanked by regions (in orange and yellow for the regions 5′ or 3′ to *nic*, respectively) that, when single‐stranded, would each bind a TraI molecule. Lower panel: Schematic representation of the OriT region of the F plasmid. The binding sites for each relaxosome components are depicted by boxes coloured according to the protein to which they bind using the same protein colour‐coding shown in the upper panel. Under each box, the protein which binds to the depicted site and the name of the site are indicated. “TraI A” and “TraI B” indicate the region 5′ and 3′ to *nic* to which the trans‐esterase and helicase domains of two individual TraI molecules bind, respectively (depicted in Fig 2B). (B) Domain structure of TraI and binding to OriT. Upper panel: Domain structure of TraI. TraI is composed of a trans‐esterase domain (orange), a vestigial (green) and an active helicase (blue) domain, and a C‐terminal domain (grey). Boundary residues are indicated for R1 plasmid TraI. Lower panel: TraI binding to OriT. TraI is indicated schematically with each volume indicating the various TraI domains using the same colour‐coding as described above. Two TraI molecules bind OriT, one on each side of the *nic* site. TraI bound to sequence 5′ (indicated by the orange strip below) to the *nic* site is bound through its trans‐esterase domain and its overall conformation is open (not shown here). TraI bound to sequence 3′ (indicated by the yellow strip above) to the *nic* site is bound through its helicase domains and its overall conformation is closed (not shown here). (C) Structure of TraI in its helicase‐loaded mode. The TraI–ssDNA complex is shown with TraI and the ssDNA in ribbon and stick representation, respectively. The domains are coloured coded as in upper panel (B). The linker between the trans‐esterase and vestigial helicase domain is shown in grey.

Relaxases are phosphodiesterases that catalyse the site‐ and strand‐specific cleavage of the plasmid OriT region at a site termed “*nic*” (Fig 2A, upper and lower panels). Upon cleavage, the enzyme remains covalently attached to the 5′ end of the T‐strand through a phosphotyrosyl linkage. It is this covalent ssDNA–protein conjugate/complex that constitutes the T4S secretion substrate that is to be transferred through the transport machinery. The transport of the relaxase alongside the T‐DNA is rationalized (and subsequently demonstrated 25) by the requirement to recircularize the single‐strand T‐DNA once the complete copy of the T‐DNA is transferred to the recipient cell.

Relaxases are usually (but not always) large multidomain proteins (Fig 2B, upper panel). In all cases, they contain a trans‐esterase (also termed “relaxase”) domain of about 300 amino acids that executes the phosphodiesterase reaction. This domain locates at the N‐terminus. Additional domains at the C‐terminus of the protein may support DNA helicase or DNA primase activities, or extra domain of unknown function 26. Relaxases can be classified into eight “MOB” families, MOB_F_, MOB_H_, MOB_Q_, MOB_C_, MOB_P_ and MOB_V_, MOB_T_ and MOB_B_ 27, among which MOB_F_ and MOB_P_ family relaxases have been the best studied 23, 24. Here, I will focus on MOB_F_ relaxases as they have been the focus of the most recent research.

MOB_F_ relaxases include TraI encoded by the F‐family plasmids (F, R1 and pED208 for example) and TrwC encoded by the R388 plasmid. Both include a helicase domain at their C‐terminus but F‐family plasmid TraI proteins have a more extensive domain structure with an N‐terminal trans‐esterase domain (residues 1–306) that catalyses the nicking and covalent attachment of the T‐strand to the relaxase 28, a vestigial helicase domain (residues 315–828) that operates as a ssDNA‐binding domain 29, an active helicase domain (residues 864–1,461) that unwinds DNA in the 5′‐to‐3′ direction, and a C‐terminal domain, the function of which is still unclear but might be used as a recruitment platform for relaxosome components 30, 31 (residue numbers here are for the R1 plasmid TraI; Fig 2B, upper panel). TrwC consists of only two domains, an N‐terminal trans‐esterase domain and a single (active) helicase C‐terminal domain 32 with functions similar to the corresponding domains in F‐family relaxases.

The functional domains of MOB_F_ relaxases appear to have different DNA‐binding requirements. The trans‐esterase domains bind with high affinity to the region of OriT immediately 5′ to the *nic* site containing sequences likely to form an inverted repeat (IR), while, as shown for F‐family relaxases, the helicase domains display sequence specificity for the region of OriT immediately 3′ to the *nic* site 29, 33. Crystal structures of the trans‐esterase domain of TrwC of plasmid R388 and of TraI of F plasmid have provided the molecular basis of the interaction between the trans‐esterase domain and its IR‐containing ssDNA‐binding site 28, 34, 35, 36. This work dating back from 2003 has been reviewed extensively and will only be described briefly here taking the TrwC trans‐esterase domain as a model 36, 37. The protein displays a fold built on a two‐layer alpha/beta sandwich, with a deep narrow cleft that forms the active site. Typically, IR repeats on double‐stranded DNA have the potential to form extruded cruciform structures, likely important for binding. In the structure, one IR arm of the extruded cruciform was used in complex formation and shown to be firmly embraced by the protein. The IR arm is followed by a ssDNA segment that enters the active site containing two catalytic tyrosines, Tyr18 and Tyr26. At this point, the ssDNA is presented with two potential exit paths 36. Tyr 18 has been shown to be the catalytic residue onto which the 5′‐phosphate resulting from the nicking/cleavage reaction in the donor cell would covalently react; Tyr26 has been implicated in a second cleavage reaction occurring, this time, in the recipient cell to create the essential 3′‐OH required for end‐joining recircularization of the plasmid DNA 38, 39. This second reaction would occur as a second copy of the *nic* site inevitably appears when a second copy of the T‐strand is “pushed” into the recipient cell (more details below). Thus, the two exit paths could be used simultaneously to bring into proximity the Y18 hydroxyl‐5′‐phosphate adduct and a free 3′‐OH resulting from the cleavage at *nic* of a second copy of the T‐strand. It is however important to note that not all relaxases are endowed with two catalytic tyrosines. When only one exists, as is the case for the TraI F plasmid relaxase, a second copy of the relaxase is required to catalyse the production of the free 3′‐OH either in the donor cell or in the recipient cell (more details below) 40.

As mentioned above, two binding sites on either side of the *nic* site provide selective binding platforms for, on the one hand, trans‐esterase binding 5′ of *nic*, and, on the other, helicase binding 3′ of *nic*. Recently, Ilangovan *et al* 41 have shown that full‐length F‐family TraI binds the ssDNA 5′ of *nic* in an open conformation being susceptible to rapid proteolysis degradation, but binds ssDNA 3′ of *nic* in a closed form being resistant to proteolytic degradation. Moreover, making elegant use of OriT‐derived oligonucleotides containing (i) a photoactivatable cleavage site instead of *nic* and (ii) judiciously positioned fluorophores, Ilangovan *et al* 41 demonstrated that OriT can simultaneously bind two TraI molecules (Fig 2B, lower panel), one on each side of the *nic* site, with the TraI bound 5′ of *nic* being in an open conformation, while that bound 3′ of *nic* being in a closed conformation, providing the first experimental evidence that, indeed, two TraI molecules can co‐occupy OriT, an observation which, as will be explained below, has profound mechanistic implications.

Ilangovan *et al* 41 also determined the structure of the closed form of TraI by cryo‐electron microscopy (cryo‐EM) to atomic resolution (Fig 2C). This structure is bound to a 22‐mer oligonucleotide derived from the sequence 3′ of *nic*. Regions of TraI for which the structure could be obtained included the trans‐esterase domain, the vestigial and the active helicase domains, but not the C‐terminal domain for which no electron density was observed, suggesting this domain is either very flexible or disordered. The salient features of the structure are the following. Firstly, while the three domains (trans‐esterase (in orange), vestigial helicase (in green) and active helicase (in blue)) are linearly arranged in the primary sequence, they are not adjacent in the three‐dimensional structure (Fig 2B and C); instead, the active helicase domain is positioned near the trans‐esterase domain, whereas the vestigial helicase domain is distal relative to the latter. Long linkers between domains facilitate such a domain configuration. Secondly, the ssDNA binds longitudinally across the entire structure, with its 5′ half bound to the trans‐esterase and active helicase domains, while its 3′ half is bound to the active and vestigial helicase domain. Interestingly, when superimposing the structure of the single IR‐bound trans‐esterase domain of TrwC with that of TraI full‐length bound to ssDNA, the DNAs sterically clash, suggesting that, in TraI, helicase‐associated ssDNA binding and trans‐esterase‐associated IR binding are mutually exclusive, possibly accounting for earlier observations of negative cooperativity between the two sites 29. Thirdly, the ssDNA is almost completely buried within the structure, explaining the remarkably high processivity of this enzyme. Indeed, TraI is one of the most processive monomeric helicases known and exhibits a fast unwinding rate of ~1,100 bp/s 42, 43. Fourthly, the vestigial and active helicase domains have very similar structures, both exhibiting the classical helicase sub‐domain organization of the SF1A/B family resembling most the RecD2 helicase, an SF1B family helicase exhibiting the same 5′‐to‐3′ directionality as TraI. Indeed, like RecD2, each helicase domain of TraI contains four sub‐domains, termed N‐terminal (N‐term), 1A, 2A and 2B. The “N‐term” domain forms an α‐helical bundle while the 1A and 2A domains both exhibit a RecA‐like fold. However, the 2B sub‐domain differs markedly from that of RecD2. Similarly to RecD2, the 2B sub‐domains of TraI, in both the vestigial and active helicase domains, are formed by sequence insertions within the 2A domain; however, the 2B sub‐domains of TraI are much larger, containing additional sequences that themselves form an additional sub‐domain termed “2B‐like”. Thus, the 2B and 2B‐like (2B/2B‐like) sub‐domains form an extended sub‐structure that is observed clamping down on top of the ssDNA, resulting in the ssDNA being mostly buried. Interestingly, in each helicase domain, these 2B/2B‐like sub‐domains are mounted onto two linker sequences that form hinges onto which these sub‐domains could pivot between two configurations, open and closed. In the open configuration, the ssDNA‐binding site of the helicase domains would be accessible to binding, and thus, the relaxase would load to the ssDNA sequence. Once bound, the 2B/2B‐like sub‐domains would close, clamping down onto the ssDNA and unwinding would start. These open/closed states of the helicase domains may or may not correspond to the open and closed states of the relaxase characterized biochemically based on the protease sensitivity experiments described above. Finally, the 2B/2B‐like domains have an additional role: they may provide the surfaces responsible for recruitment of TraI to the T4S system. As will be described below, VirD4, a T4S system protein, serves as a recruitment platform for the relaxosome. Because of this role, VirD4 is often known as “the coupling protein”. Two regions, termed TSA and TSB, of R1 plasmid TraI (an F‐family plasmid) were identified to serve as translocation signals, mediating presumably the recruitment of TraI to the T4S system, perhaps through interactions with the VirD4/TraD protein 44. Similar sequences were identified in the R388 TrwC relaxase 45. Remarkably, these translocation signals are not located at either the C‐ or N‐terminus of these proteins as is usually the case for most known translocation signals, but in their middle. TSA and TSB map to the 2B/2B‐like sub‐domains and the putative VirD4‐interacting region within these sub‐domains map opposite to the ssDNA‐interacting region, suggesting that these regions are available for binding to the T4S system, even when bound to ssDNA 41, 46.

### Accessory proteins

The relaxase is part of a bigger complex, the relaxosome, which contains 2–3 additional proteins, termed “accessory” proteins (Fig 2A, upper panel). Much has been written about these proteins (reviewed in refs. 10, 41), and thus, only a short description will be given here for the F plasmid relaxosome accessory proteins. In this case, the relaxosome is formed of the relaxase TraI, two plasmid‐encoded proteins, TraM and TraY, the genome‐encoded IHF (integrated host factor) heterodimeric protein, and OriT. IHF consists of two ~10‐kDa subunits, about 30% identical in sequence. The structure of IHF bound to a double‐stranded DNA shows that IHF induces a 160° bend in the DNA 47. TraY is a small protein (131 residues), structurally related to the ribbon‐helix‐helix (RHH) family, which bends the DNA by 50–55° 48. F TraM is a 127‐residue protein with an N‐terminal domain that binds DNA, and a C‐terminal domain responsible for tetramerization. Its N‐terminal DNA‐binding domain homodimerizes to form a RHH, and two TraM tetramers are required to cooperatively bind a minimal DNA‐binding site 49.

These proteins together with the relaxase bind multiple sites within OriT (summarized in ref. 50 and in Fig 2A, lower panel). In the OriT of the F plasmid (F OriT), there are two IHF‐binding sites (IHF A and B), two TraY‐binding sites (sbyA and sbyC), three TraM‐binding sites (sbmA‐C), and as mentioned above, two TraI‐loading sites on each side of *nic*. The site sequence is the following: sbmA, sbmB, IHF B, sbmC, sbyA, sbyC, IHF A, TraI‐binding IR (TraI‐A), *nic* and TraI helicase loading site (TraI‐B). The relaxosome proteins assemble on the OriT DNA in a defined order 51, 52 and distort its topology severely and locally, leading to disruption in its supercoiled and double‐stranded states. Although the interactions of these proteins with DNA has been extensively studied (reviewed in refs. 10, 24, 37), very little is known about how these proteins interact with each other. However, multiple reports have shown that relaxase activity is stimulated in the presence of accessory proteins, indicating direct interactions between these proteins 33, 52, 53, 54. As a matter of fact, everything points to the relaxosome being an extremely complex structure: (i) some of these proteins have stable oligomeric states, but some others appear to adopt various oligomerization states upon binding DNA; and (ii) some of these proteins are able to bend DNA quite severely, implying that DNAs and proteins apparently distant from each other based on the linear organization of the various binding sites in OriT might in fact be within proximity. Solving the three‐dimensional structure of a relaxosome is one of the greatest challenges of conjugation research.

How the relaxosome is recruited to the T4S system apparatus itself has been investigated extensively (reviewed in ref. 55). All interactions are with VirD4, the coupling protein, itself an integral part of the T4S system 56. TraM of F interacts with the C‐terminal tail of VirD4/TraD, an interaction that was visualized crystallographically 57. TraI exhibits two translocation signal sequences, but whether these sequences bind VirD4 directly has not been demonstrated (see above). Finally, accessory proteins TrwA (a potential homologue of TraM) and TrwC (the relaxase) from the R388 plasmid interact with VirD4/TrwB 58. Recently, more details of the interaction between a VirD4 protein, that of *Legionella pneumophila*, with accessory proteins and chaperones have been revealed 59. These details are reminiscent of the TraM/TraD complex by Lu *et al* 57 as the interactions are also with the C‐terminal tail of VirD4.

## The T4S system

Early in conjugation research, it became apparent that the genes involved in conjugation could be divided in two sets: the mating pair formation (*mpf*) genes responsible for pilus biogenesis and mating junctions, and DNA‐transfer replication (*dtr*) genes responsible for processing the DNA at OriT 60, 61. The MPF complex is now known as the type IV secretion system while the DTR components are known as the relaxosome. Linking the two complexes is the VirD4 coupling protein (CP), which recruits the relaxosome and presents it to the T4S system. As it seems that VirD4 might be an integral and constitutive part of the T4S system 56, I will include it in the description of the system, instead of treating it separately. Also, because each individual VirB1‐11/VirD4 protein has been the subject of exhaustive reviews 62, 63, I will focus on describing the large subassemblies and assemblies that the Gram‐negative bacterial T4S systems form, the structures of which have been unravelled recently.

T4S systems are unique among secretion systems in being able to transport both proteins and DNAs. Functionally, they cumulate many transport and assembly functions: (i) they function as pilus biogenesis machines able to construct and retract pili made of thousands of pilus subunits; (ii) they act as DNA transporters; and (iii) they act as protein transporters. Conjugative T4S systems are remarkable as being able to cumulate all three functions in one apparatus. No wonder the T4S system architecture is immensely more complex than any other secretion systems in Gram‐negative bacteria (see a review by Costa *et al* 11 for an extensive review of secretion systems in these bacteria and another by Galan and Waksman 64 on injection machines, a more focused review targeted to a larger audience).

The first large subassembly of a T4S system (that encoded by the pKM101 plasmid) was described in 2009 when Fronzes *et al* 13 published the purification and subsequent characterization of its structure by cryo‐EM. This complex, named “the core complex” was 1.05 megadaltons in size and composed of 14 copies of three of the VirB proteins, VirB9, VirB10 and the lipoprotein VirB7. Being embedded in the two membranes of *Escherichia coli* (VirB10 is indeed seen making a channel in the outer membrane 65 and has a trans‐membrane helix inserted in the inner membrane), this complex was thought to form the T4S system channel. This notion prevailed for a number of years until Low *et al* 14 published a larger subassembly of the T4S system, this time encoded by the R388 system (a system closely homologous to that of pKM101). This larger complex is composed of eight VirB components, VirB3‐10. It contains the same core complex of VirB7, VirB9 and VirB10 (renamed outer‐membrane (OM) core complex (OMCC) since it is primarily directed towards the OM) but this complex is observed mounted via a stalk structure on an IMC made of 12 copies each of VirB3, VirB4, VirB5, VirB6, VirB8 and the 14 N‐terminal trans‐membrane helices of VirB10 emanating from the OMCC. Remarkably, the VirB4 ATPase forms two hexameric barrels protruding in the cytoplasm. This architecture of a head (the OMCC) mounted on two legs (the two VirB4 hexameric barrels) via a stalk/neck was unprecedented among bacterial secretion systems since all others were best described as concentric stacks of rings extending from the cytosol to the extracellular milieu.

When describing T4S systems, it is convenient (albeit imperfect) to categorize them in two classes: A and B. T4AS systems broadly consist of the 12 VirB1‐11/VirD4 proteins (one exception is the F‐family T4AS system which contains more). T4BS systems are generally much larger, consisting of many more proteins, for example 27 for the Dot/Icm T4S system from *L. pneumophila* 8, 66. Although the *cag* T4S system from *H. pylori* was initially classed as a T4AS system, it seems more related to the T4BS systems class. Nevertheless, T4BS systems include most of the VirB/VirD4 proteins of T4AS systems; for example, all three archetypal T4AS ATPases, VirB4, VirD4 and VirB11, have homologues in the *Dot/Icm* system (DotO, DotL and DotB, respectively) or the *cag* system (CagE, Cag5 and Cagα). Interestingly, in the T4AS system encoded by the F plasmid, VirB11 is absent. Thus, overall, T4AS systems can be seen as “minimal” T4S systems with T4BS systems being expanded and more elaborated versions. Below, I will review the structures of OMCCs and IMCs of T4AS and T4BS systems, pointing to the differences between the two classes and also their common features.

### The outer‐membrane core complex (OMCC)

**Figure 3:**
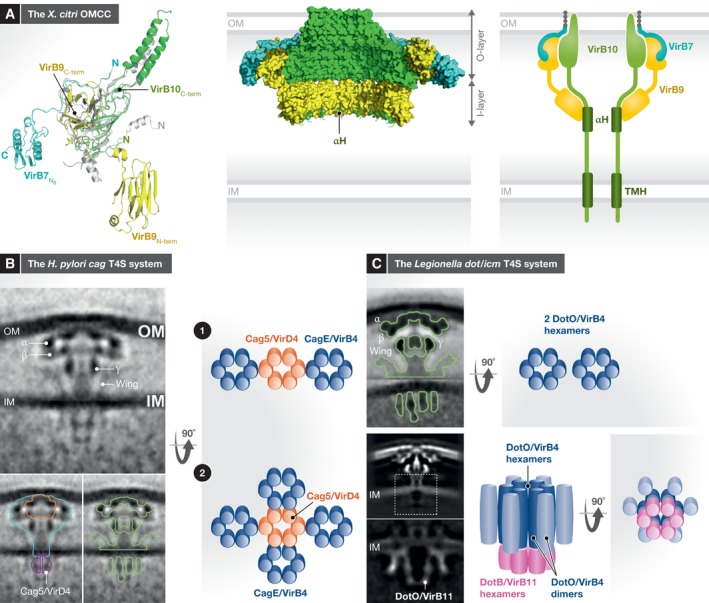
The architecture of the T4S system from *X. citri*,* H. pylori* and *L. pneumophila* (A) The *X. citri* OMCC. Left panel: Superposition of the heterotrimeric unit of the *X. citri* OMCC structure (composed of full‐length VirB7, VirB9, and VirB10) with the heterotrimeric unit of the O‐layer of the pKM101 OMCC (made of the full‐length VirB7, and the C‐terminal domains of VirB9 and VirB10). Structures are shown in ribbon representation. *X. citri* proteins are color‐coded in green, yellow and cyan blue for VirB10, VirB9, and VirB7, respectively. The pKM101 O‐layer heterotrimer is shown in grey. Domains of *X. citri* as well as some of the N‐ and C‐termini are labeled. A striking feature of all high resolution OMCC structures is the presence of long inter‐domain linkers, which project each domain of VirB9 and VirB10 a long distance away. Middle panel: Surface of the full‐length *X. citri* OMCC. Color‐coding is as in left panel. For the VirB10 N‐terminal domain, only density for an α‐helix (labeled “αH”) was observed and a model corresponding to residues 150 to 161 was derived. Seven of the 14 heterotrimeric units are shown so as to access a view of the interior of the OMCC. This interior is lined with VirB10 as also observed for the O‐layer of the pKM101 OMCC [65]. The I‐ and O‐layers are indicated. Right panel: Schematic diagram of the OMCC. αH (residues 150‐161 of the N‐terminal domain of VirB10) is the only secondary structure in the N‐terminal domain of VirB10 that is observed in the electron density of the *X. citri* OMCC. However, there are 149 residues N‐terminal to this region that are not observed, including a trans‐membrane helix (TMH) that inserts into the IM. 14 of those were hypothesized to form a channel in *Legionella*. (B) The *H. pylori cag* T4S system. Left upper panel: A slice through the side view of the composite sub‐tomogram average. Averages aligned on the periplasmic and cytoplasmic parts are stitched together using the IM as the boundary. Some regions of the density mentioned in the text are indicated and labelled. Left lower two panels: Two duplicated side views as in left upper panel are shown so as to compare the size and location of various other structures. The orange outline indicates comparison of the R388 OMCC to the *cag* T4S system structure; the blue outline indicates the position of the purified *cag* T4S system OMCC within the *cag* tomography structure; the magenta outline indicates the predicted location of the coupling protein Cag5/VirD4 based on the structure of the DotL/VirD4 homologue; the green outline indicates the *Legionella dot/icm* T4S system structure superimposed on the *cag* T4S system structure. Right panel: Schematic diagram of the IMC ATPases. The four “tubes” of density observed in the sub‐tomogram average together with the central density observed might correspond to either two side‐by‐side CagE/VirB4 hexamers flanking one Cag5/VirD4 hexamer (upper panel) or four CagE/VirB4 hexamers surrounding one Cag5/VirD4 hexamer (lower panel). VirB4 and VirD4 subunits are represented as cylinders colour‐coded blue and orange, respectively. (C) The *Legionella dot/icm* T4S system. Upper left and right panels: The *Legionella* T4S system observed by cryo‐ET by Ghosal *et al* (2017, 2018) and interpretation of the IMC ATPase organization 69, 73. Upper left panel: A slice through the side view of the composite sub‐tomogram average of the *Legionella dot/icm* T4S system. Some regions of the density mentioned in the text are indicated and labelled. Upper right panel: Schematic diagram of IMC ATPases. The four “tubes” of density are interpreted as projections of two side‐by‐side DotO/VirB4 ATPases. Lower left and right panels: The *Legionella* T4S system observed by cryo‐ET by Chetrit *et al* 15. Lower left panels: The top panel shows a slice of the sub‐tomogram average of the entire T4S system while the bottom panel focuses on the IMC. In the IMC, four “tubes” of density are clearly visible as for all T4S systems visualized by cryo‐ET, but, in this study, the two central tubes are bound to two additional “tubes” of density corresponding to the DotB/VirB11 ATPase. Lower right panels: Schematic diagram of the IMC ATPases: it is suggested that the four “tubes” of density are projections of a hexamer of DotO/VirB4 dimers with the DotO/VirB4 subunits involved in hexamerization stacked against the DotB/VirB11 hexamers (two views are shown here: one side view and the other 90° away).

In most T4AS systems investigated so far, the OMCC forms a cage with two layers, the O‐ and I‐layers. It is made of VirB7, VirB9 and VirB10, with VirB10 lining the interior of the cage and forming a channel in the OM while also being inserted in the IM (Fig 3A). The VirB10 ring is buttressed on the exterior by binary complexes of VirB7 and VirB9. Most OMCC structures (pKM101, R388, *A. tumefaciens*,* H. pylori* cag and *Xanthomonas citri*) exhibit 14‐fold symmetry 13, 16, 65, 67, 68; the only exception is that of *L. pneumophila*, which exhibits 13‐fold symmetry 15, 69.

The atomic resolution structure of the O‐layer has been known for some time and has recently been confirmed (65, 70; reviewed, e.g., in refs. 9, 66, 71). In the OMCC O‐layer of both pKM101 65 and *X. citri* 70, the O‐layer is made of the C‐terminal domains of VirB9 and VirB10 and the full‐length VirB7, a lipoprotein playing a role in OM insertion 13. The parts of the structures encompassing the VirB9 and VirB10 C‐terminal domains as well as the N‐terminal first 16 residues of VirB7 perfectly superimpose (Fig 3A), indicating a striking conservation of structures and frameworks among T4S system OMCCs in spite of very different functions (the *X. citri* T4S system is a protein transporter, while the pKM101 system is involved in conjugation). The salient features of the O‐layer structure are as follows: (i) VirB10 forms an alpha‐helical channel in the OM (more specifically, 14 alpha‐helical hairpins come together to form a circa 35 Å opening through the OM), (ii) VirB10 lines the interior of the O‐layer, (iii) VirB10 inserts in both the IM and the OM, and (iv) the C‐terminal domain of VirB10 connects with its N‐terminal domain located in the I‐layer by a very long, transversal, 28 residues linker which projects the N‐terminal domain 57 Å away from the C‐terminal domain. In *X. citri*, an additional feature of the O‐layer is an additional C‐terminal domain in VirB7 that forms 14 satellites around the OMCC. Recent work has suggested that deletion of the alpha‐helical hairpins does not affect secretion 67.

The atomic resolution structure of the I‐layer has however only recently been determined 70. In this study, because the full‐length OMCC of the bacterial killing T4S system from *X. citri* was solved at high resolution, an atomic model of the I‐layer could be derived for the first time, confirming some of the features of an equivalent cryo‐EM structure from the pKM101 system obtained at a lower resolution 72. The I‐layer is made of 14 copies of the N‐terminal domains of VirB9 and VirB10. However, in the structure by Sgro *et al* 70, the N‐terminal domain of VirB10 was disordered and therefore could not be traced except for a short α‐helix (Fig 3A). The salient features of this structure are the following (Fig 3A). Firstly, the existence of a long linker between domains in VirB10 is confirmed and an equally long linker (19 residues) is observed between the N‐ and C‐terminal domains of VirB9; as a result, the N‐ and C‐terminal domains of VirB9 are 26 Å apart. Secondly, there are very few interactions between the O‐ and I‐layers, suggesting that the O‐ and I‐layers may acquire various relative orientations, possibly rotating independently of each other within limits afforded by the long linkers between the N and C‐terminal domains in both VirB9 and VirB10. The N‐terminal domain of VirB10 being disordered in that structure, no further insight from the description already provided by Rivera‐Calzada *et al* 72 could be provided. In this lower resolution cryo‐EM structure, density for VirB10 could be identified based on its presence in the full‐length pKM101 OMCC and its absence in a variant pKM101 OMCC lacking this domain: the domain was shown to have an extended structure of three successive α‐helices interspaced by long linkers 72 (Fig 3A).

T4BS systems OMCCs retain the core OMCC structure of the T4AS system but are much larger, being made of more components (Fig 3B and C). The *H. pylori cag* OMCC is composed of five proteins, two of them larger homologues of VirB9 and VirB10: CagM, CagT, CagX/VirB9, CagY/VirB10 and Cag3 16, 68. This OMCC was visualized by negative‐stain EM as well as cryo‐electron tomography (cryo‐ET) and shown to exhibit 14‐fold symmetry 16, 68. The *Legionella* system is composed of at least five proteins (DotC, DotD, DotF, DotG and DotH) with DotD, DotH and DotG possibly larger homologues of VirB7, VirB9 and VirB10, respectively 15, 69, 73. The *Legionella* cryo‐ET OMCC structures are 13‐fold symmetrical. The OMCCs of both *H. pylori* and *Legionella* are remarkably similar, displaying additional densities compared to the minimal T4AS OMCC, termed α, β, γ and wings on the side (Fig 3B and C). A fifth patch of density linking the β and γ densities in *Legionella* was termed the elbow [preprint: 69]. Based on negative‐stain images of the *H. pylori* OMCC where either Cag3 or CagT was missing from the complex, these two proteins were assigned to the α and β densities, respectively. By elimination, the γ density was ascribed to CagM, although this would need to be verified. In *Legionella*, the situation might be more complex and the additional densities might be ascribed to several proteins: DotC forms the top part of the γ density; DotH forms the central part of β, the bottom part of γ and the elbow; DotD connects from the OM (a similar role as T4AS VirB7) but also forms the peripheral region of β. DotG has a C‐terminal domain (residue 844–1,045) clearly recognizable as a homologue of the C‐terminal domain of VirB10 (the domain identified in the pKM101 structure of the O‐layer as forming the OM channel) but its N‐terminus is much larger (843 amino acids) and includes a ~600 residues repeat region likely to fold into long β‐helices. The cryo‐ET structures of the *Legionella* T4S system clearly show a periplasmic channel at the base of the OMCC and reaching out to the IM 15, 69. The channel is formed by DotG (perhaps its β‐helix domain) 15. DotF forms the wings. Other proteins such as DotK, IcmX, DotA and IcmF are also part of the OMCC and could be located to some of the densities mentioned above. Of particular interest, the channel appears plugged on top and this plug appears to be primarily formed of IcmX and also IcmF.

### The inner‐membrane complex

**Figure 4:**
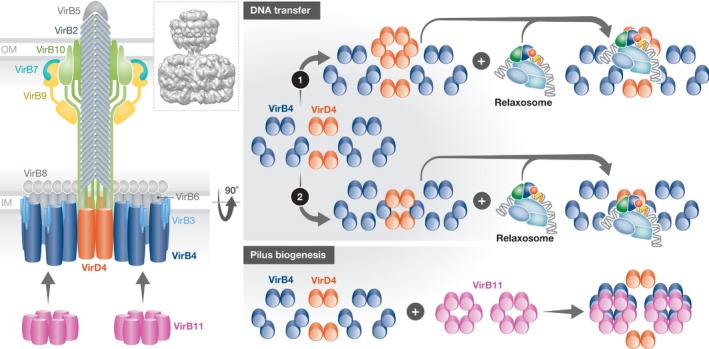
Architecture of the R388 conjugative T4S system Left panel: Schematic diagram of the R388 VirB3‐10/VirD4 structure by Redzej *et al* 56. The VirB4/TrwK ATPases are shown as trimers of dimers in blue while the VirD4/TrwB ATPases are shown as dimers, consistent with the observation of the electron density for that complex. Inset: side view of the density map of the negative‐stain EM structure of the VirB3‐10/VirD4 by Redzej *et al* 56. Right panels: Schematics of IMC ATPases. The structure shows two VirB4/TrwK trimers of dimers forming the two barrel‐like densities and two VirD4/TrwB dimers linking them. These are shown as blue and orange yellow circles, respectively. This apparatus can operate in two modes: in a pilus biogenesis mode upon binding of VirB11/TrwD to the two VirB4/TrwK ATPases, or in a substrate‐transfer mode upon binding of the relaxosome to the VirD4/TrwB. I hypothesize that VirB11 acts as a “hexamer organizer” remodelling each VirB4/TrwK trimers of dimers into active hexamers in order to execute pilus biogenesis, while the relaxosome induces remodelling of VirD4 dimers into hexamers (option 1) or mixed VirD4 and VirB4 dimers into hexamers (option 2). In option 1, the resulting VirD4 hexamer is positioned sideways; in option 2, the hexamer is central, just underneath a potential VirB10 channel and the pilus. The relaxosome is represented as in Fig 2A.

The first glimpse of a T4AS system IMC was provided by Low *et al* (2014) when a large 3‐megadalton complex including the VirB3‐10 proteins encoded by the R388 plasmid was purified and visualized by negative‐stain EM (Figure 4) 14. This complex has been extensively reviewed 9, 63, 74 and will only be briefly described here. The major features of this complex are the following. Firstly, it is made of 12 copies of each VirB3, VirB4, VirB5, VirB6 and VirB8, and 14 copies of the N‐terminal IM‐inserting segments of VirB10. Secondly, it is composed of a periplasmic part named “arches”, connecting to an IM‐inserted region, followed by two barrel‐like structures in the cytosol. The cytosolic part is twofold symmetrical with the two barrels made of two VirB4 hexameric ATPases. Thirdly, no central channel was observed, suggesting that any substrate might pass unfolded through one of the ATPases. Recent evidence have demonstrated that the relaxase indeed needs unfolding to pass through conjugative T4S systems 75.

**Figure 5:**
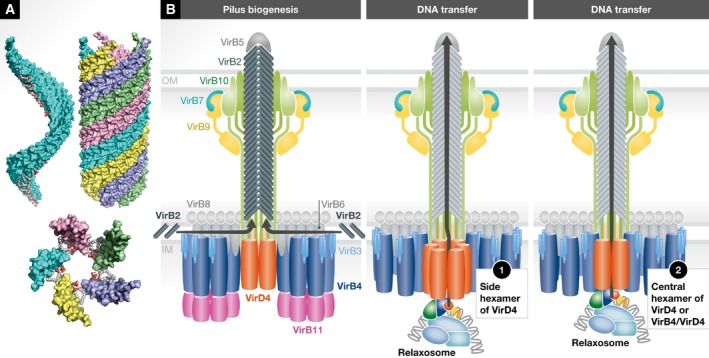
The F‐family pilus and mechanism of T4S (A) The structure of the F‐family pilus. Upper left panel: One array of VirB2 pilus subunits with VirB2 shown in light blue surface representation and the phospholipid shown in sphere representation colour‐coded in white and red for carbon and oxygen atoms, respectively. Upper right panel: Five arrays of VirB2 subunits shown as in the upper left panel except that the five arrays are colour‐coded in a different colour. Lower panel: The pentameric base of the F pilus. Representation and colour‐coding are the same as in upper panels. (B) Mechanism of pilus biogenesis and substrate transfer by conjugative T4S systems. Conjugative T4S systems can operate in two modes: a pilus biogenesis mode (left) and a DNA‐transfer mode (right). VirB11 hexamer binding reshapes VirB4 to switch the T4S system to its pilus biogenesis mode. In that mode, VirB2 pilus subunits are extracted by VirB4, perhaps using a “lateral gate” mechanism to capture pilin subunits. The lateral gate mechanism was first described to account for the mechanism of the SecYEG transport apparatus 107. In the DNA transfer mode, the relaxosome is hypothesized to induce either hexamerization of VirD4 ATPase dimers to form a VirD4 homo‐hexamer situated on the side (option 1) or the formation of mixed VirB4/VirD4 hexamers located centrally, just under the VirB10 channel and the VirB2 pilus (option 2). The white arrows indicate the transfer route for each option. The T4S system and the relaxosome are as in Fig 4.

A subsequent study where an additional element of the R388 T4AS system, the VirD4/TrwB coupling protein, was purified together with the VirB3‐10 complex shed further insights on a more complete T4S system 56 (Fig 4). In the VirB3‐10/VirD4 complex structure, two independent dimers of VirD4/TrwB are observed, located almost transversally relative to the two VirB4 hexameric barrels (Fig 4). Given that VirD4 operates as a hexamer, it is clear that in this structure, VirD4 is not complete. Transition from a dimer to a hexamer would be required for function and this was hypothesized to occur upon recruitment of the relaxosome (Figs 4 and 5). Another potential mechanism for a VirD4 transition from a non‐functional state to a functional one would be the formation of mixed hexamers with VirB4. Indeed, VirD4 and VirB4 have strikingly similar structures 76, 77, 78, 79. Moreover, in both the VirB3‐10 structure by Low *et al* 14 and that of VirB3‐10/VirD4 by Redzej *et al* 56, the VirB4 barrels are distinctly formed of trimers of dimers with the dimers appearing to interact loosely with one another. Thus, it is not far‐fetched to hypothesize that VirD4 might form mixed hexamers with VirB4, a transition that would facilitate the handover and transition of the T4S system from a protein transporter mediated by VirB4 to a DNA transporter mediated by VirD4. Alternatively, the formation of VirB4/VirD4 mixed dimers positioned centrally within the system might be sufficient to execute ssDNA transport (Figs 4 and 5); in that case, a mixed‐composition ATPase would locate centrally, facilitating transfer to the centrally positioned OMCC and pilus (see below).

The T4BS system IMC was recently visualized at low resolution (30‐40 Å) by cryo‐ET and provided previously uncharacterized overall features of T4S systems *in situ* 15, 16, 69, 73 (Fig 3B and C). The *H. pylori cag* IMC is remarkable in that it appears to confirm the results by Redzej *et al* that T4S systems contain many ATPases at their base 16, 56 (Fig 3B). Indeed, the sub‐tomogram average established at a very low resolution of 39 Å could be best fitted to simulated structures that included four longer side barrels and a central shorter one. The identity of these barrels was not established but the shorter barrel was speculatively assigned to Cag5/VirD4, an assignment that remains to be proven. The *Legionella* IMC architecture was reported in two independent publications that although they report very similar tomogram sections, the results are interpreted in very different ways. Ghosal *et al* 73 reported the first *in situ* structure of the *dot/icm* system and described an IMC very similar to the one observed in conjugative T4AS systems: a two‐barrelled IMC where the barrels are made of the VirB4 ATPase, a result that appears to be confirmed in a more detailed study published recently (Fig 3C). Chetrit *et al* 15 reported a more complete structure on the same system where the location of the DotB/VirB11 ATPase could be located. Indeed, both studies show that *Legionella* tomograms display four density tubes corresponding to two VirB4 barrels (two tubes of density would arise from the 2D projection of one ATPase). But, in the structure by Chetrit *et al* 15, however, the cytosolic end of the two central density tubes is seen to be connecting with two additional tubes of density attributable to DotB/VirB11. Thus, DotB/VirB11 appears to be straddling the two VirB4 barrels. This led the authors to completely reinterpret the symmetry of the IMC. Indeed, VirB11 is a constitutive hexameric ATPase 80, 81, 82, and therefore, given its central location in the IMC part of the tomogram, sixfold symmetry for the entire IMC was applied. The sixfold averaged structure indicates an IMC with astonishing features: (i) the DotB/VirB11 hexamer sits on top of a hexamer of DotO/VirB4, and (ii) each of the six subunits in this DotO/VirB4 hexamer is bound to a second subunit of DotO/VirB4, suggesting that DotO/VirB4 is an hexamer of dimers, an unprecedented observation in AAA^+^ ATPase 83. How two different hexameric ATPases, DotB/VirB11 and DotO/VirB4, might work together being physically linked in a stack? There are examples of AAA^+^ ATPases that contain two stacking ATPase domains in one single polypeptide, and it is therefore possible that a stack of two distinct ATPases would work similarly 83. Also, how a hexamer of dimers with two different subunit–subunit interfaces, one for hexamerization and one for dimerization, would be prevented from aggregating into infinite arrays remains to be determined. Nevertheless, if confirmed, such an architecture would have the advantage of producing a continuous central channel through the stacked ATPases and then through a DotG/VirB10 channel in the OMCC (see below).

### The stalk and the arches/wings

The stalk is the structure that links the OMCC and the IMC (Figs 3 and 4). In the negative‐stain EM structure by Low *et al* 14, the stalk appears to be made of pillars, the origin of which is unclear. Also, this structure is obstructed at the IM, likely an artefact of the negative‐stain. It was hypothesized that the stalk structure could be a prepilus onto which the pilus could be elaborated. The N‐terminal sequence of VirB10 between the IM‐embedded trans‐membrane segment and the structured periplasmic N‐terminal domain is rich in proline and therefore likely to be unstructured; this part was hypothesized to form a drape all around the stalk allowing direct access for substrate from the periplasm to the OMCC secretion chamber 72, as is the exceptional case for secretion of effector molecules by the T4S system from *Bordetella pertussis*. A similar secretion chamber was described for the *Legionella dot/icm* system [preprint: 69]. In the latest *Legionella* tomography work, a stalk is also visible, made by DotG/VirB10, and forms an uninterrupted funnel/channel 15, 69. Whether this channel continues through the IM is not clear but possible since DotG/VirB10 inserts in the IM. In *H. pylori*, a similar “funnel” like structure is also apparent, but it appears to be obstructed 16.

How such a complex machinery made of an OMCC, a stalk and an IMC assembles in the first place remains a mystery, but a consensus has emerged that assembly starts at the outer membrane. This was first suggested by the fact that the OMCC assembles spontaneously 13, indicating that the OMCC is likely to form first and serve as a “seed” for IMC assembly, a suggestion backed up by earlier evidence 84, 85 and also evidence published recently 15, 69.

## The T4S system pilus

All conjugative T4S systems of Gram‐negative bacteria elaborate a pilus. While *H. pylori* produces appendages clearly dependent on the *cag* T4S system, the *Legionella dot/*icm system does not appear to. For decades, the pilus elaborated by the F T4AS system was the only visible structure and its presence helped define the so‐called *mpf* genes (now termed “T4S system genes”) involved in pilus biogenesis. Pili are helical polymers of the major pilus subunit (or pilin), VirB2, but also contains a minor subunit, VirB5 (reviewed in refs. 86, 87, 88). The roles of the pilus are the subject of debate. However, the following is by now broadly accepted: (i) conjugative pili are involved in recipient or phage recognition. Indeed, pili are the first point of contact with recipient cells or phages. Pilus‐mediated cell–cell interactions in bacteria are mediated by adhesins, proteins often located at either the tip or the side of pili 89. A report has localized VirB5 at the tip of the T‐pilus encoded by the Ti plasmid from *A. tumefaciens* 18. However, little is known about specific receptors that would serve as a target for a conjugative pilus adhesin. OM protein A (OmpA) and the lipopolysaccharides (LPS) are known to play a role (reviewed in ref. 61 and for more recent studies 90) but direct interactions between these molecules and a conjugative adhesin have not been demonstrated. (ii) Long and flexible types of conjugative pili such as the F pilus are retractable. It has been known for some time that conjugating bacteria form tight and extensive junctions, with the pilus mediating the first contacts and then retracting to facilitate cell‐to‐cell contacts (reviewed in ref. 61; see also refs. 20, 21, 91). Retractability has been directly visualized by Clarke *et al* 19 by live‐cell imaging using fluorescently labelled F pilus‐specific phages. This work shows that the pilus constantly expands and retracts in a stochastic fashion. While pilus biogenesis requires energy, it has been suggested that retraction does not and occurs spontaneously 92. (iii) The F‐family pili are made of 1:1 molar ratio of VirB2:phospholipid units and are large enough to accommodate ssDNA and unfolded relaxase transfer. The F pilus indeed forms a 5‐start helical array with an internal lumen of 28 Å in diameter 93 (Fig 5A). All pilus subunits (pilins) first locate in the IM. They are extensively processed (reduced from three to two TM segments and acetylated for F‐family pili; or circularized for T and P pili from Ti and P plasmids, respectively) before being extracted from the IM to form an extracellular helical filament during pilus biogenesis by the T4S system (reviewed in ref. 86). In that process, for F‐family pili, it is a specific 1:1 protein:phospholipid complex that is extracted and thousands of such complexes are assembled into a pilus. In the pilus, the phospholipid head groups line the lumen, neutralizing basic residues otherwise involved in protein–lipid interactions inside the IM. Neutralization of charges within the lumen would facilitate ssDNA transport. The presence of lipids within the pilus may also facilitate reinsertion of pilin:phospholipid complexes back into the IM during retraction, perhaps explaining why retraction might be energy independent; it may also facilitate insertion within the recipient membranes to allow injection of the ssDNA/protein substrate. (iv) During pilus biogenesis, pilus subunits are added from the base and the pilus is likely to go through the centre of the OMCC to cross the OM 94. (v) Conjugative pili appear to be randomly placed over the entire cell surface. In F‐family pili (including those produced by the F, pED208 or R1 plasmids), there are 5 (F) to 20 (pED208) pili per cell that appear randomly distributed over the entire cell surface. This is puzzling because (i) plasmids are located at precise positions corresponding to quarter or midpoint of the cell and (ii) T4S systems appear to be either helically positioned along the long cell axis or at the cell poles 91, 95, 96. Perhaps even more puzzling is that once a T4S system machinery has produced a pilus, if it is not engaged in transport, it appears to disassemble rapidly as no machinery appears visible at its base (Banerji and Waksman, unpublished and 16). This is particularly striking in the recently reported case of the *H. pylori* T4BS system‐dependent filaments, where a few examples were given where no T4BS system was observed at the base of the filament but instead observed some distance away 16.

## Mechanisms of conjugative transport

T4S systems can function in two different modes, all encapsulated in one single machinery in the case of conjugative systems: (i) a pilus biogenesis mode and (ii) a substrate‐transport mode. Within the substrate‐transport mode, the machinery must cater for the transport of a mixed substrate formed of a ssDNA covalently bound to an unfolded peptide (the relaxase) 75, 97, i.e. two different types of macromolecules that have profoundly different chemical and steric requirements for transport. Executing such diverse functions within a single apparatus is bound to require profound remodelling of the machinery at various stages during the process, together with sophisticated and timely regulatory mechanisms enabling transitions at both macroscopic and microscopic levels.

### Pilus biogenesis

Pilus biogenesis by T4S systems is poorly understood. The best mechanistically characterized system for pilus biogenesis is that of the chaperone–usher pathway, but the study of these systems was greatly facilitated by the small size of the apparatus (just one circa 85‐ to 100‐kDa protein embedded in the OM, the usher), and the fact that it could be reconstituted *in vitro* 98, 99. Unfortunately, T4S systems are extremely large, and therefore cannot be purified in a functional form, and cannot be reconstituted *in vitro*. Two T4S system ATPases appear to be involved in pilus biogenesis, VirB4 and VirB11 100, 101, 102, 103. VirB4 interacts with VirB2 and evidence suggests that VirB4 functions as a dislocation motor to extract pilins from the IM during T4S system‐mediated pilus biogenesis. VirB11 appears to function together with VirB4 to induce a structural change in the pilin and thus was suggested to modulate VirB4 dislocase activity. Interaction between VirB11 and VirB4 have been documented and from the work by Chetrit *et al* 15 and others, it is reasonable to hypothesize that VirB11 might stack against VirB4, thereby executing a transition in VirB4 from non‐functional trimers of dimers as seen by Low *et al* 14 or Redzej *et al* 56 to active hexamers (Figs 4 and 5B). Note that the F plasmid T4S system does not contain a VirB11 homologue; in that case, VirB4 might form constitutive hexamers. How VirB4 may orchestrate pilus biogenesis may only be speculated. It is reasonable to hypothesize that the interactions of VirB4 and VirB2 are through their TM segments. ATP hydrolysis would then extract pilin–phospholipid complexes from the membrane, depositing it onto an unknown structure which might be present in the stalk that would serve as a prepilus. Two VirB4 hexamers might act in concert to execute fast VirB2‐phospholipid assembly into a 5‐start helical array (Fig 5B). Defining the details of pilus biogenesis by T4S systems is one of the major challenges facing the field.

### Substrate transport

Once a pilus has been produced, the T4S secretion system either disassembles or captures a relaxosome to form a pre‐initiation complex. This pre‐initiation complex remains dormant until the pilus is engaged with a recipient cell, at which point conjugation begins. Most likely, the relaxase is transported first, piloting the ssDNA through the machinery. Thus, the T4S system operates as both a protein and DNA transport machinery. Given the radically different chemical nature of these two biological macromolecules, it is likely that the secretion pathway for protein transport is distinct from the secretion pathway for ssDNA transport. I would therefore like to suggest that during active conjugation, the T4S system switches from a protein‐transport machinery to a ssDNA‐transport one once protein transport is completed. Thus, conjugative T4S systems would exist in three different states executing three different functions through three distinct secretion pathways: pilus biogenesis, unfolded relaxase transport and ssDNA transport; and thus would need to operate two switches during their lifetime: one from pilus biogenesis to unfolded relaxase transport and another from unfolded relaxase transport to ssDNA transport.

This unique ability to execute three different functions may explain why T4S systems require so many ATPases: extracting pilins from the IM requires active engagement of a powerful ATPase, likely VirB4 102; unfolding of the relaxase would require the application of large mechanical forces only afforded by another powerful ATPase, VirB11 perhaps 80, 104; finally, DNA might be threaded through the system through the “massaging” of a ssDNA‐threading ATPase, perhaps VirD4 35, 77. It also explains the extreme complexity of the T4S system architecture, with multiple hexameric ATPase barrels in the IMC, stacked ATPases, sometimes the lack of a central channel, and some other times the presence of one 14, 15, 16, 56, 73.

Because of the multiple processes sometimes taking place simultaneously during conjugation, the mechanism of conjugation is by and large still unclear, although recent breakthroughs have shed light on some of its aspects. An attempt at incorporating established and novel knowledge to describe how conjugation might unfold is described below. Various steps and scenarios are illustrated in Fig 5B.

1. *Formation of the pre‐initiation complex*. Zechner *et al* 24, 37 have argued that prior to the start of conjugation, a pre‐initiation complex consisting of a relaxosome bound to a T4S system lies pre‐formed, ready to burst into action as soon as an external stimulus is generated by contact with a recipient cell. At this point, the relaxase is engaged with the plasmid supercoiled DNA through its trans‐esterase domain. This domain is able to nick the T‐strand, but the relaxosome does not transition to its “relaxed” form because the 3′ end is tightly held within the active site, and therefore, the DNA undergoes constant nicking/end‐joining at the *nic* site without change in the overall topology and conformation of the complex. Further, although DNA binding of accessory proteins alters somewhat the local topology of OriT, it does not do so sufficiently to generate a ssDNA stretch 3′ of *nic* to allow helicase loading. At this stage, the relaxosome is bound to the coupling protein VirD4, possibly through interactions of VirD4 with the translocation signal sequences of the relaxase and/or with some of the accessory proteins (see details above). I hypothesize that upon binding the relaxosome, homo‐hexamerization of VirD4 or hetero‐hexamerization of VirD4/VirB4 is induced (Figs 4 and 5B). At this stage, I also assume that the T4S system is directly connected to a pilus, likely encased within the OMCC, and thus, a pilus, a resting T4S system and a resting relaxosome are interacting with each other, in an assembly that is poised to act.
2. *Activation by recipient cells, retraction of the pilus, formation of the tight conjugative junction, and activation of the relaxosome*. In the presence of recipient cells, conjugation is induced. The signal is likely to be triggered by the pilus adhering to some receptors in the recipient membranes. Such receptor could be OmpA, LPS or a yet‐unidentified molecule 90. Also, the T4S adhesin protein responsible for receptor binding has not yet been identified, VirB5 being a potential candidate 18, 105. Pili of the F‐family have a natural propensity to expand and retract, and thus, binding of the pilus to the recipient cell would inevitably bring donor and recipient cells together until cell‐to‐cell contact is made to form a tight junction that seems to favour conjugative transfer. However, conjugation is also observed when cells are some distance from each other 22. At this point, the relaxosome can be “safely” activated. Indeed, timing is of the essence: firing DNA processing before recipient cells are presented to the donor cells would be wasteful and counterproductive. Activation of the relaxosome may consist in (i) nicking of the *nic* site and release of the resulting 3′ end together with covalent attaching of the relaxase to the resulting 5′‐phosphate; and (ii) formation of a ssDNA bubble 3′ to the *nic* site to facilitate helicase loading. This helicase activity might be contributed by a cellular helicase in the case of relaxases that do not have a helicase domain OR might be contributed by a second copy of the relaxase for relaxases that have a helicase domain in addition to a trans‐esterase domain 41. Once the helicase is loaded, it starts unwinding DNA and pumping ssDNA through the T4S system. However, an integral part of the activation process must also be the priming of the relaxase molecule covalently bound to the T‐strand's 5′‐phosphate for transport, its unfolding and its engagement with the T4S system. Here, there are three scenarios depending on whether it is the protein or the ssDNA that is transported first or whether they are transported simultaneously. To my knowledge, this is not firmly established; yet, the implications are significant. The prevalent notion so far is that the protein “pilots” the ssDNA or drags it along 25; thus, protein transport may occur first. If that is the case, then ssDNA bubble formation and helicase loading would need to occur AFTER protein transport. Whichever order in which the relaxase or the ssDNA are transported, this raises the following general question: Whereas the DNA‐processing steps prior to ssDNA transport are well known, what about protein processing? This is an issue that needs to be addressed: Relaxase unfolding is required 75, but how does this occur? To date, no chaperone involved in maintaining the relaxase in an unfolded or semi‐unfolded state has been identified; the ATP‐driven unfoldase remains unknown; the steps leading to relaxase priming and insertion within the unfoldase remain to be characterized.
3. *Transport through the machinery and transition from a protein‐transport machinery to a ssDNA‐transport machinery or vice versa*. At this point in time, two architectures of the T4S systems have been proposed. One was observed by negative‐stain EM on purified materials and also observed *in situ* by cryo‐ET 14, 16, 56, 69, 73 and consisted of multiple hexameric ATPase barrels, some laterally located 14, 69, 73, and some both laterally and centrally located 16, 56 (Figs 3B and C, and 4). The other was observed *in situ* by cryo‐ET 15 and consisted of stacked ATPases, one of them a hexamer of dimers (Fig 3C). While the protein‐transport pathway is unknown, that of the ssDNA has been investigated by Christie and colleagues in a fascinating investigation which makes use of a transfer DNA immuno‐precipitation (TrIP) assay 101, 106. The T‐DNA was shown to translocate through the *A. tumefaciens* VirB/D4 T4S system by forming close contacts sequentially with the VirD4 coupling ATPase, the VirB11 ATPase, the IMC proteins VirB6 and VirB8, the OMCC VirB9 and the pilus subunit VirB2. VirB4 does not contact the ssDNA but coordinate transfer from VirB11 to VirB6 and VirB8. The involvement of VirB2 would indicate a role for the pilus in ssDNA transport. However, this pathway does not fit with any of the architectural frameworks known for T4S systems; this is exciting because it points to an architecture of the T4S system different to those described so far. Indeed, in the recently proposed ATPases stack model 15, VirD4/DotL is absent and VirB4/DotO would contact the substrate since it is stacked with VirB11. In the Low *et al* 14 and Redzej *et al* 56 models, VirB11 is absent, but whatever its position within the system, it would make contact with the ssDNA first before VirD4 or VirB4 since it can only stack against an already positioned VirD4 or VirB4 ATPase or a T4S system component, or bind on the side of the apparatus (an unlikely proposition). Finally, in the TrIP results, VirB10 does not contact DNA; yet, VirB10/substrate interaction is suggested by the tomography work on the *H. pylori* and *Legionella* systems, where VirB10/DotG as well as VirB10/CagY appears to form the OMCC central channel, and by the X‐ray crystallography work on the pKM101 system, where VirB10/TraF lines the interior of the OMCC. Thus, one would need to envisage a different architecture to account for the TrIP data, a state which will undoubtedly be unravelled in the near future, emphasizing again the great versatility and plasticity of the T4S system.
4. *Transfer to a recipient cell and termination of conjugation*. While details of the transfer pathways through the T4S system are painstakingly being collected, mechanistic details for the transport through the recipient cell membrane is lacking. Most importantly, the channel through the recipient cell's membrane is unknown. The mechanisms by which ssDNA or protein are transferred across the OM and IM of the recipient cell therefore remain to be established.
5. Much better documented is the mechanism by which conjugation terminates. As mentioned above, conjugation terminates in the recipient cell by an end‐joining reaction that is executed by the same relaxase molecule that is being transported unfolded and covalently attached to the ssDNA. In that model, the relaxase would refold once transported and would immediately travel along the ssDNA in the 5′‐to‐3′ direction as the ssDNA is pumped through. As a complete version of the ssDNA is fed to the system, a nicked 3′ end would inevitably appear at some point during transfer, which will serve as substrate for an end‐joining reaction by the relaxase. This nicked free 3′ end is the one generated by the very same relaxase before it got transferred through the apparatus; thus, the same relaxase nicks the T‐strand in the donor cell, covalently attaches to the resulting 5′‐phosphate, is transferred to the recipient cell and travels along the ssDNA that is emerging in the recipient cell until the 3′ end presents itself and end‐joining occurs resulting in a circular ssDNA that undergoes replication. This model is only valid if the nicked 3′ end generated by the nicking reaction in the donor cell is not extended by rolling circle DNA replication in the donor cell. If it is, then a second nicking reaction needs to occur either in the donor cell or in the recipient cell. This second reaction might be executed by a second relaxase either in the donor cell or in the recipient cell. Indeed, DNA‐independent transport of the relaxase has been observed 25.

## Conclusions

In this review, I have attempted to mix the old to the new and account for decades of intense research efforts on a process of fundamental importance, both historically and biomedically. The discovery of conjugation led to a succession of seminal discoveries that provided profound insights into genome biology, gene organization, structure and regulation. Conjugation is the principal means by which genes (including antibiotic‐resistance genes) are horizontally transferred from one bacterium to another and is therefore a major contributor to bacterial genome plasticity, evolution and adaptation. Remarkably, after more than seven decades of research in the field, much is still to be discovered (see Box 1). This is not entirely surprising given the extreme complexity of the process, the size of the machine and its uncanny ability to execute many different functions, from pilus biogenesis (the assembly of helical filaments from thousands of identical protein:phospholipid binary complexes), to unfolded relaxase transport, and ssDNA transfer, the complication of the last two exacerbated by the fact that both are covalently linked! A recent exciting development has been the use of cryo‐ET in unravelling the outline and shape of these complex machines, adding another valuable method to the arsenal available to researchers. It is however a blend of high‐resolution (X‐ray crystallography and cryo‐EM) and low‐resolution (cryo‐ET) techniques allied to clever biochemical and molecular biological techniques that will win the day. As always, multi‐disciplinary efforts are the sure route to success and in that respect, there is every reason that the mechanism of type IV secretion in its amazing diversity and complexity will be one day elucidated.

## Conflict of interest

The author declares that he has no conflict of interest.

**Box 1:In need of answers**

- What is the architecture and structure of the relaxosome?
- How does a fully assembled relaxosome interact with a fully assembled T4S system?
- What is the role of the pilus: an adhesive device for recipient cell recognition or/and a conduit for the relaxase–ssDNA conjugate?
- What is the nature of the mating signal?
- How does donor–recipient cell contact trigger relaxosome DNA nicking and unwinding?
- How is the unfolded relaxase primed for transport? What is the unfoldase and what is the protein translocase
- What is the secretion pathway for the relaxase–ssDNA conjugate? Are they the same or are there two pathways, one for protein transport and one for ssDNA transport? How is one handed over to the other?
- What is the nature of the tight conjugative junctions?
- What is the mechanism of pilus biogenesis by conjugative T4S systems?
